# Clusthaplo: a plug-in for MCQTL to enhance QTL detection using ancestral alleles in multi-cross design

**DOI:** 10.1007/s00122-014-2267-1

**Published:** 2014-01-31

**Authors:** Damien Leroux, Abdelaziz Rahmani, Sylvain Jasson, Marjolaine Ventelon, Florence Louis, Laurence Moreau, Brigitte Mangin

**Affiliations:** 1 Unité de Mathématique et Informatique Appliquées de Toulouse, INRA, UR875, Chemin de Borde Rouge, 31326 Castanet-Tolosan, France; 2 EURALIS SEMENCES, Service Biométrie, Domaine de Sandreau, 31700 Mondonville, France; 3 Syngenta Seeds, 12 chemin de l’Hobit, 31790 Saint-Sauveur, France; 4 INRA, UMR 0320 / UMR 8120 Genet Vegetale, Ferme du Moulon, 91190 Gif Sur Yvette, France

## Abstract

*****Key message***:**

**We enhance power and accuracy of QTL mapping in multiple related families, by clustering the founders of the families on their local genomic similarity.**

**Abstract:**

MCQTL is a linkage mapping software application that allows the joint QTL mapping of multiple related families. In its current implementation, QTLs are modeled with one or two parameters for each parent that is a founder of the multi-cross design. The higher the number of parents, the higher the number of model parameters which can impact the power and the accuracy of the mapping. We propose to make use of the availability of denser and denser genotyping information on the founders to lessen the number of MCQTL parameters and thus boost the QTL discovery. We developed clusthaplo, an R package (http://cran.r-project.org/web/packages/clusthaplo/index.html), which aims to cluster haplotypes using a genomic similarity that reflects the probability of sharing the same ancestral allele. Computed in a sliding window along the genome and followed by a clustering method, the genomic similarity allows the local clustering of the parent haplotypes. Our assumption is that the haplotypes belonging to the same class transmit the same ancestral allele. So their putative QTL allelic effects can be modeled with the same parameter, leading to a parsimonious model, that is plugged in MCQTL. Intensive simulations using three maize data sets showed the significant gain in power and in accuracy of the QTL mapping with the ancestral allele model compared to the classical MCQTL model. MCQTL_LD (clusthaplo outputs plug in MCQTL) is a versatile and powerful tool for QTL mapping in multiple related families that makes use of linkage and linkage disequilibrium (web site http://carlit.toulouse.inra.fr/MCQTL/).

**Electronic supplementary material:**

The online version of this article (doi:10.1007/s00122-014-2267-1) contains supplementary material, which is available to authorized users.

## Introduction

Since the work of Lander and Botstein (1989), the detection and the mapping of loci affecting quantitative traits (Quantitative Trait Loci or QTL) using genetic markers have led to a number of interesting results in the dissection of the genetic architecture of complex traits. For decades, especially in plant populations where genetic crosses can be easily controlled, the QTL analyses were largely conducted within large bi-parental family. However, as highlighted by Blanc et al. (2006), the use of multiple descendants and particularly multiple connected populations allows to explore a larger allelic diversity and to address complex behavior of the QTL within different genetic backgrounds.

The first attempt to model QTL effect in multiple connected or related populations was by Rebai and Goffinet (1993) in a diallel design within a frequentist framework. The QTL was assumed to be a fixed effect and a diallel modeling served for its allelic effects. This type of model was further extended to any multi-cross design in Jourjon et al. (2005) by the use of a so-called connected model that assumes that the allelic effect of the founders of the multiple connected populations is identical over the populations. In this framework, the marker regression approach (Haley and Knott 1992) allows a robust linear model for QTL detection and a fast computation task while having asymptotically all the qualities of a maximum likelihood approach (Rebai et al. 1995). Multiple QTL models can be studied using cofactor-based methods, MQM (Jansen 1994), MIM (Kao et al 1999) or iQTLm algorithm (Charcosset et al. 2001). Within the Bayesian framework, a hierarchical modeling allowing to analyze any known pedigree was proposed nearly simultaneously for multi-allelic QTLs (Yi and Xu 2001) or biallelic ones (Bink et al. 2002). However, the MCMC algorithm that is computationally demanding and difficult to prune is necessary to detect and estimate multiple QTLs. Despite its simplicity, the use of the identical-by-descent (IBD) status of the QTL alleles between descendants, that was proposed by Xie et al. (1998) within the mixed model framework for independent families, was the last to be adapted to connected populations in Crepieux et al. (2005). In this framework, the single QTL variance is estimated using ASREML (Gilmour et al. 1995). However, extension to multiple QTLs is not fully developed.

A way to enhance the feasibility of QTL fine mapping is to combine linkage mapping with linkage disequilibrium analysis of the founders of the multi-cross designs. Several statistical methods that include population genetics concepts to model the evolution of the linkage disequilibrium between markers and the causal mutation appear simultaneously in combined methods of linkage disequilibrium and linkage analysis (LDLA mapping). Meuwissen et al. (2002) used the evolution model to predict the IBD probabilities of the parents at the QTL and plugged these probabilities within the mixed linear model for linkage mapping. Perez-Enciso (2003) used the same evolution model in a full Bayesian framework. In a frequentist framework, Farnir et al. (2002) proposed a model based on the Wright–Fisher evolution of the QTL and marker allelic frequencies that was plugged within the usual mixture model of linkage analysis. However, software applications of these above methods were largely developed for the half-sib design of animal breeders and were not adapted to the plant breeding designs.

In parallel, new populations that allow to enhance QTL fine mapping have been developed as the maize nested association mapping (NAM) population (Yu et al. 2008) or the *Arabidopsis Thaliana* multi-parent advanced generation intercross (MAGIC) (Kover et al. 2009). However, until recently, the NAM population has been analyzed with the joint linkage model which has a mean family parameter and intra-family hierarchical QTL parameters (Li et al. 2011) giving as many QTL parameter as founders. The MAGIC population has also been analyzed with one QTL parameter per founder. These models did not take advantage of shared IBD alleles of the founders that should be predicted using the linkage disequilibrium and thus did not combine linkage mapping and linkage disequilibrium analysis.

With the lower cost of genotyping, all the descendants of a multi-cross design could be genotyped for a highly dense marker map, even if this will cause high redundancy information due to the small expected number of recombinations. With highly dense map, the inference of the QTL alleles by linkage within a cross is no longer necessary or it can be approximated by a simple imputation on missing marker genotypes. So, the data can be analyzed with the unified mixed model of association (Yu et al. 2005). In this model, the QTL is assumed to be bi-allelic and identity-by-state alleles at the QTL position are assumed to be identical over all the populations. This is the most parsimonious model but it has been showed that it is not always the most powerful model and that complex traits should be analyzed by different multi- or bi-allelic QTL models to capture the complexity of allelic variation (Bardol et al. 2013).

MCQTL (Jourjon et al. 2005) is a software application dedicated to QTL mapping in multi-population design. It implements the tools of the frequentist framework (the marker regression approach (Haley and Knott 1992), the multiple QTL detection by iQTLm algorithm (Charcosset et al. 2001), the threshold by permutation (Doerge and Churchill 1996). It has been applied to the dissection of the architecture of a number of traits these two recent years (see Cadic et al. (2013) for sunflower, Fournier-Level et al. (2013) for *A. Thaliana*, Lagunes Espinoza and Julier (2013) and Moreau et al. (2012) for *Medicago*, Pauly et al. (2012) for ray-grass, Lariepe et al. (2012) for maize, among others). In the MCQTL model, the number of model parameters is directly related to the number of parental alleles. The goal of the MCQTL_LD extension is to reduce the number of these parameters. This goal is important from a statistical point of view, since the power of a statistical test is inversely related to the number of parameters. Fortunately, it is a reasonable goal from a genetics point of view, since plant breeding populations were created from a small base of ancestors, so only a small number of ancestral alleles is segregating in these populations.

To achieve this goal, we developed clusthaplo, an R package (R Development Core Team 2008), that permits to group the parent lines of multi-cross designs using a genomic similarity that reflects the probability of sharing the same ancestral allele. Computed using a sliding window along the genome and followed by a classical method of clustering, the genomic similarity allows the local clustering of the parents. Our assumption is that the parents belonging to the same class transmit the same ancestral allele. So their putative QTL allelic effects can be modeled with the same parameter, leading to a parsimonious model that should be powerful and accurate. We also extended MCQTL (Jourjon et al. 2005), a stand-alone Java and C++ software application that runs on Linux operating system, to allow it to be fed by clusthaplo outputs. This extension was named MCQTL_LD and it was developed to lessen the number of model parameters following the clustering computed by clusthaplo.

To reach conclusions on the effective interest of the parent clustering, intensive simulations were conducted on real multi-cross designs, reflecting the extent of variation between a design composed with few large families and a design composed with many small families.

## Method

The MCQTL connected model is the marker regression model (Haley and Knott 1992) with genetic cofactors, previously detected QTL for example, and a scanned putative QTL. The genetic effects of QTL and cofactors are assumed to be identical over the families which corresponds to a genetic assumption of no interaction between allelic effect and genetic background.

Let *d* denote the descendant family of two parent lines *i*, *j*, the phenotypic value *Y*_*dn*_ of the *n*th individual in this family is modeled by

$$Y_{dn} = \mu_{d} + \sum_{l=1}^L \sum_{ij} p^l_{dn,ij} ( \alpha^l_{i} + \alpha^l_{j} ) + \epsilon_{dn}$$

where *μ*_*d*_ is the global mean of the descendant family *d*, *L* − 1 is the number of genetic cofactors, *p*_*dn*,*ij*_^*l*^ is the probability of the *dn*th individual having genotype *ij* at the QTL or cofactor locus *l* given marker information, *α*_*i*_^*l*^ and *α*_*j*_^*l*^ are the additive effects of the *i*th and *j*th parent at locus *l*, and $\epsilon_{dn}$ the residual error. The model is presented as a purely additive QTL model but a more complex model involving dominance is implemented in MCQTL.

Clusthaplo is an R package (R Development Core Team 2008). It is designed to perform clustering of haplotypes that ought to share a ancestor. It is based on a pairwise similarity measure computed for every pair of haplotypes using a sliding window along the genome. For the markers inside a window, we build a weighted graph having the haplotypes as nodes and the pairwise haplotype similarities as edge weights. Then, the haplotype clustering for the position at the center of the window is obtained by building the transitive closure of the filtered graph. We propose two different methods for the graph filtering step. One is based on Hidden Markov model (HMM) and the second on computing a threshold by simulations.

After the clusthaplo analysis, at each scanned locus *l*, each parent *i* is assigned to a cluster *cl* that is assumed to be an ancestral allele. Let *f* ^*l*^ be the function that assigns the parent lines at their corresponding cluster at position *l*, i.e. *f* ^*l*^(*i*) = *cl*. The MCQTL_LD model is obtained by plugging the function in the MCQTL model leading to

$$Y_{dn} = \mu_{d} + \sum_{l=1}^L \sum_{ij} p^l_{dn,ij} ( \alpha^l_{f^l(i)} + \alpha^l_{f^l(j)} ) + \epsilon_{dn}$$

**Fig. 1:**
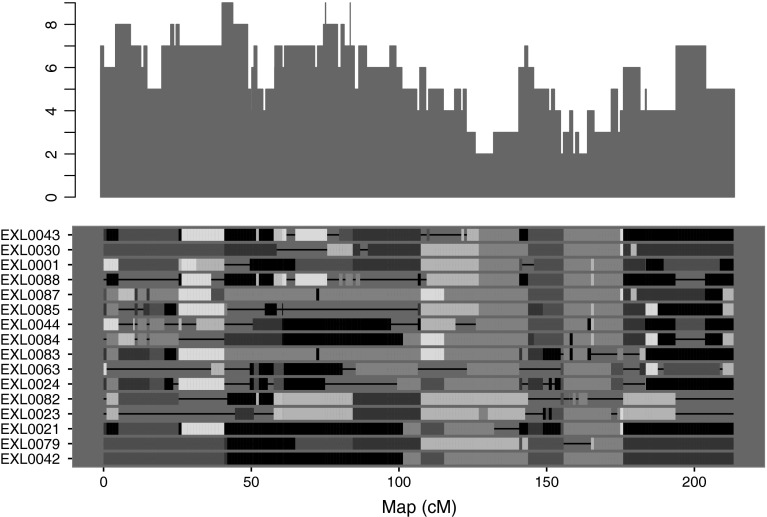
Clusthaplo outputs along a chromosome. *Top*: number of estimated ancestral alleles at each locus, *bottom*: *color-coded* representation of the chromosome of the 16 studied parent lines (in row) at each locus. Each distinct ancestral allele is given a single color when it is shared by at least two haplotypes, otherwise a thick *black line* is drawn. Note that the occurrence of the same color at two different loci does not imply anything on the relatedness of the corresponding alleles (color figure online)

Clusthaplo outputs are illustrated in the Fig. 1 which presents an example along a chromosome with the number of ancestral alleles found at each locus and the clustering of 16 parent lines.

The Fisher test is the most usual test for QTL detection in a linear model. However, due to the local clustering of parent lines which changes along the genome, the degrees of freedom of the Fisher test change from place to place.

The implication in an MCQTL_LD detection scan is that QTL will have more chance to be detected on loci with a local clustering with few classes. To avoid this problem, we changed the QTL detection test in MCQTL. Instead of comparing the Fisher tests between scanned positions, we compared their *p* values. We transformed the *p* value with the −log_10_() function for practical and readability reasons.

Clusthaplo and MCQTL can be run using, each, a different marker map. That of clusthaplo is the one used to genotype the parent lines and is generally a high-density map. That of MCQTL is the genetic consensus map that allows the joint QTL linkage mapping of the multiple families.

To link both analyses, the two marker maps are aligned, so at least two markers per chromosome are mandatory to anchor the map alignment. In case of multiple anchors they have to be roughly colinear between the two maps. The QTL scan positions along the genome are defined by the consensus map and a fixed progression step as per MCQTL. The scanned positions are the loci where clusthaplo computes the similarity score between the parent haplotypes and provides a clustering of them. Clusthaplo outputs XML files suited for MCQTL_LD.

The only change to perform an MCQTL_LD analysis is to add in the parameter file an XML tag specifying the names of these XML files.

### Li and Jiang’s similarity score

Li and Jiang (2005) proposed a new haplotype similarity measure that generalizes several haplotype similarity measures already published. It combines through weight functions the number of alleles alike-in-state between a pair of haplotypes, and the length of their longest common genome segment.

Let *t* be a particular locus of the genome and the center of the sliding window. We denote by *h*_*i*_^*t*^ and *h*_*j*_^*t*^ the part of two haplotypes *h*_*i*_ and *h*_*j*_ that belongs to the sliding window, and by *h*_*i*_*k*_^*t*^ with $k \in [1,K]$ the alleles of the haplotype *i* at the *k*th markers within the sliding window (respectively, for haplotype *h*_*j*_). Assuming that the genetic or physical marker map is known, let *x*_*k*_^*t*^ be the distance of the marker *k* to the locus *t*. Then, the Li and Jiang’s similarity score at the locus *t* is:

$$s^t_{i,j}= \sum_1^K w_1(x_k^t) I( h_{i_k}^t, h_{j_k}^t ) + \sum_{k=l'}^{r'} w_2(x_k^t)$$

where *I*(*a*,*b*) = 1 if allele *a* and *b* are alike-in-state and *I*(*a*, *b*) = 0 otherwise. *w*_1_(*x*) and *w*_2_(*x*) are two non-increasing weight functions (except for *w*_2_ in the vicinity of *x* = 0 since Li and Jiang (2005) imposed *w*_2_(0) = 0 to resemble to a length measure previously defined) and *l*′ and *r*′ are the marker indexes located at the left and the right of the locus *t* that mark the boundary of the longest common segment within the sliding window.

Notice that we slightly modified the Li and Jiang’s similarity score to be able to compute the similarity at a locus *t* which is not necessarily a marker position. This is motivated by the use of this similarity score during the whole genome scan for QTL detection that can be performed anywhere on the genome.

As highlighted by Li and Jiang (2005), due to the two weight functions, the similarity score definition is very flexible. It gives a score that is robust against recent marker mutations and genotyping/haplotyping errors. It is also able to apprehend partial sharing from a ancestral haplotype in common due to historical recombination events.

Let *s*_map_^*t*^ be the maximum value of the similarity score in the window *t* which is equal to the similarity of an haplotype with itself (*s*_*i*,*i*_^*t*^) assuming no missing data. The similarity score is normalized to the interval [0, 1] using *s*_map_^*t*^ so it does not increase with the length of the sliding window and the number of markers within the window, leading to $\tilde{s}^{t}_{i,j}= \frac{s^{t}_{i,j}}{s^{t}_{{\rm{map}}}}.$

### Extended Li and Jiang’s similarity score

While it presents good properties, the major flaw of the similarity score is that it offers poor reliability when it is calculated upon regions with too few markers. When the sliding window does not include enough markers, it is necessary to get the missing information about the haplotype relatedness from another source. Hence, we extended the Li and Jiang’s similarity score to incorporate prior knowledge on the relatedness of the haplotypes. If the pedigree of all haplotypes is known, the kinship coefficient can be a good information to incorporate in the score. When the pedigree is unknown, this coefficient can be estimated using all the markers in the genome.

The new similarity score $S^{t}_{i,j} \in [0,1]$ is computed using both kinship and marker information. Let $K_{i,j} \in [0,1]$ be the kinship coefficient between the haplotypes *i* and *j*, $\tilde{s}^{t}_{i,j}$ the normalized Li and Jiang’s similarity score at the scanned position *t* and $P^t \in [0,1]$ a weight function depending on locus *t*, the new similarity score is defined by:

1 $$S^{t}_{i,j}= P^t \times \tilde{s}^{t}_{i,j} + (1 -P^t ) \times K_{i,j}$$

While the Li and Jiang’s similarity score is varying with the locus *t*, the kinship is an average estimate of the genetic correlation between the haplotypes that does not depend on *t*. Since the kinship does not stress the similarity variations along the haplotype, the Li and Jiang’s similarity score is considered as the most accurate estimation of the local similarity between haplotypes when enough marker information is available.

The *P* ^*t*^ function rates the reliability of the Li and Jiang’s similarity score. When the similarity score is highly reliable, we want *P* ^*t*^ ≈ 1 so $S^{t}_{i,j} \approx \tilde{s}^{t}_{i,j},$ otherwise when the Li and Jiang’s similarity score is highly unreliable, since computed on very few markers, *P* ^*t*^ should be close to 0 and so *S*_*i*,*j*_^*t*^ ≈ *K*_*i*,*j*_.

Li and Jiang’s similarity score is judged reliable when computed on a window with a high density in markers. We suggest to use the similarity score *s*_map_^*t*^ as a measure of this reliability. Indeed, when both the weight function *w*_1_(*x*) and *w*_2_(*x*) are constant, the marker density within the sliding window is proportional to *s*_map_^*t*^. Moreover, as *w*_1_(*x*) and *w*_2_(*x*) are assumed to be non-increasing functions, for a given density within the window, *s*_map_^*t*^ increases as the markers are closer to the locus *t*. So, the similarity score *s*_map_^*t*^ is a good candidate to rate the reliability of the Li and Jiang’s similarity score and its capacity to estimate a local similarity.

Let *s*_map_^*^ = max_*t*_ *s*_map_^*t*^ be the overall maximum value of the similarity score along the genome. Hence, we choose to measure the reliability of Li and Jiang’s similarity score $P^t \in [0,1],$ with respect to the test position *t*, by:

2 $$P^t=\frac{s^{t}_{\rm{map}}}{s^{*}_{\rm{map}}}$$

*P* ^*t*^ is the function of the markers map, the test position *t*, the length of the sliding window and the choice of the weight functions *w*_1_(*x*) and *w*_2_(*x*).

Given the Eqs. (1) and (2), the new similarity score $S^{t}_{i,j} \in [0,1],$ with respect to the test position *t*, is:

3 $$S^{t}_{i,j}= \frac{s^{t}_{\rm{map}}}{s^{*}_{\rm{map}}} \times \tilde{s}^{t}_{i,j} + \left(1 - \frac{s^{t}_{\rm{map}}}{s^{*}_{\rm{map}}}\right) \times K_{i,j}$$

This similarity score is a generalization of the Li and Jiang’s similarity score. It ensures an optimal use of the available information: the prior knowledge contained in the kinship coefficients and the local similarity score with respect to each test position. Moreover, the use of the *P* ^*t*^ reliability score introduces an accurate balance in the use of both scores. However, when a part of the genome is very densely marked, the parts of the genome that are considerably less dense get a reliability weight close to zero. The similarity scores between haplotypes are then close to their kinship coefficients and so do not reflect the local similarity. To avoid this type of problem, we sightly transform the extended score by computing the maximum of *s*_map_^*t*^ only on sparse windows. The *P* ^*t*^ reliability function is computed using only the sparse density windows and let equal to 1 within the non-sparse windows. The required number of markers within the sliding window cuts the windows in these two groups. It is a parameter controlled by the user. The kinship coefficients can be provided by the user. However if they are not, the default kinship coefficient between two haplotypes is the alike-in-state measure using the whole genome marker information.

There are different weight functions implemented in clusthaplo for *w*_1_ and *w*_2_. The simplest are the 1 and 0 constant functions. The other functions are probability density functions that are adapted to the window length in such a way that the total weight of the window is 0.95. The Exponential density function was chosen because the linkage disequilibrium between two loci decreases at an exponential rate at each generation. Its *λ* parameter is calculated to have 0.475 probability on the window positive part. Then, the function is symmetrized leading to 0.95 for the whole window. The Laplace density function that has the same form but does not put a weight equal to 1 at the middle of the window is implemented and its *λ* parameter is used to fit the function to the window length. The Gaussian density function with its variance parameter and the uniform density function with its support parameter are also implemented.

### Clustering the haplotypes

Clustering of the haplotypes is performed at each locus along the chromosome by building the transitive closure of a filtered weighted graph which is a method similar to the threshold IBD model of Bink et al. (2012). At each locus, we build a complete connected graph the nodes of which are the haplotypes. Its edges are weighted by either the similarity score of the haplotype pair or their (0, 1) state obtained by HMM that is described below. Given a threshold, the filtering step is performed by removing the edges with a weight below the threshold or a 0 state in the HMM. Then the transitive step is performed by putting in the same class all the haplotypes that are still linked in the graph. The number of classes is varying along the genome from 1 (all haplotypes in the same class) to the initial number of haplotypes (no clustering). Each class of haplotypes is then assumed to transmit the same ancestral allele.

### Hidden markov models

For a pair of haplotypes, we can look at the observation series *S*_*i*, *j*_^*t*^ for $t=1,\ldots,T$ as the outputs of an HMM with hidden states 1 when the pair of haplotypes shares the same ancestral allele and 0 when it does not. Assumptions for a HMM are not fulfilled by the observation series *S*_*i*,*j*_^*t*^ . Indeed the conditional independence of *S*_*i*,*j*_^*t*^ given the hidden state is correct if the windows used to compute the similarity measure do not overlap which is not the case for a sliding window. Moreover, the stationary assumption that the transition from the hidden state 0 to the hidden state 1 (reciprocally from 1 to 0) does not depend on *t* is correct only if the locations *t* are regularly spaced. This can be slightly false since the test positions in clusthaplo are computed given a fixed step progression but are forced to be located at each marker of the MCQTL consensus map. Despite these illicit assumptions, we performed a number of HMM analyses and found that they gave very interesting classification results. However, we observed that a two-state HMM gave sometimes very poor fit compared to models with three or four states. So, we decided to fit the data with a HMM having two, three or four hidden states and to choose the best model according to their BIC score. Whatever the model chosen, the output of the HMM analysis is 0 or 1 for an haplotype pair at each locus. The loci having the state with the highest similarity scores are put in the 1 class, the others in the 0 class.

### Threshold computation methods

Two methods using intensive simulations are proposed to compute a threshold that controls the risk of deciding that two haplotypes are related though they are not. Each method proceeds in the same way. A set of haplotypes is simulated and the similarity scores for all the pair of haplotypes are computed using the same marker map, scanned loci and window length as the analysis on the initial data set. The process is repeated a number of times and all the computed score values are pooled to form an empirical distribution that mimics the null distribution of the similarity scores for a set of independent haplotypes. This empirical null distribution is used to find the empirical quantile associated to a given type I error. The two methods differ on how they simulate the unrelated or independent haplotypes.

### Equilibrium sampling

The equilibrium sampling simulates random haplotypes assuming that all the markers are at equilibrium with equifrequencies of their alleles.

The equilibrium assumption could lead to a inaccurate threshold when the marker density is very high and particularly when a lot of markers share the same locus. Indeed, with more and more markers in equilibrium sharing the same locus, the similarity score between two random haplotypes has a high probability to be equal to zero, so the null distribution is largely skewed toward zero, and the (say 95 %) quantile is then small, resulting in too much clustering of the haplotypes.

### Mosaic sampling

The equilibrium assumption is too strong with dense marker map and the assumption of equifrequency is always wrong. However, in most case, the limited number of haplotypes does not permit to get an accurate estimate of allele frequencies. Moreover, haplotypic blocks should be considered in a dense map. The mosaic sampling is the method proposed to solve these two problems. The mosaic method mimics the evolution of an isolated population from founders that are the set of initial haplotypes. With no mutation and no migration, the allele frequencies do not change. With a long-time evolution, the isolated population tends to be composed of nearly unrelated individuals if the number of initial founders is high. We model the crossing-over by the usual Poisson law. Assuming the independence of the crossing-overs along the genome and along the genealogy, the numbers of breaks in a chromosome of length *L*_*c*_ Morgan during *N*_*G*_ generations is a Poisson law of parameter equal to *N*_*G*_ *L*_*c*_. This law is used to sample the number of breaks per chromosome. The break positions are sampled using an uniform law along the chromosome. Then, each block between breaks (considering that the start and the end of the chromosome are special breaks) is formed by the marker information of an equivalent block randomly drawn from the founders. This sampling process is repeated to generate a new set of haplotypes that are a mosaic of initial founder haplotypes.

### Treatment of missing marker data

Clusthaplo provides three ways of dealing with missing data, false, true or non-assigned options. During the computation of the similarity score, when the comparison between the allele carried by each haplotype at a given locus involves a missing data, the result of the comparison is always false, the locus weight is not added to the similarity, true, the locus weight is added, or non-assigned, the locus weight is not added. So, for the part of the similarity score concerning the alike-in-state allele comparison, the false and non-assigned options give the same result. The limits of the longest common segment between two haplotypes are defined by successive identical alleles around the center of the window. Differences between the false and non-assigned options occur when missing data are encountered at the center of the window, the false option gives a longest common segment of null length whereas the non-assigned option begins the longest common segment at an informative position close to the window center.

### Material

Three maize (*Zea mays* L.) data sets served to compare QTL detection using MCQTL to the same analysis using the clusthaplo plug-in and MCQTL_LD. These three sets represent three contrasted designs from a small number of large-size families to a huge number of medium-/small-size families:

1. The Syngenta design was a complete half-diallele of four parent lines with six F3 families of large size. There were in average 144 genotyped individuals per family (minimum size 141, maximum size 148). The family consensus map had a marker density of about one marker per 3.1 cM, 514 markers in total for a map of 1,584 cM. One SNP every 1.65 cM in average was used to genotype the parent lines.
2. The Euralis2007 design was a partially connected design with 12 parent lines and 8 F2 families of size ranging from 60 to 182 (124 in average).
3. The Euralis2005 design was a connected design of 16 parent lines and 21 families of both F2 and F3 types (18 F3 families and 3 F2 families). A total of 928 observed individuals were obtained with small- to medium-size families (minimum size 21, maximum size 87).

For the Euralis designs, the family consensus map had 511 markers with a marker density of about 1 marker every 4 cM. The genetic map of the parent lines had 4,005 markers with an average one marker every 0.5 cM. The three designs, the crosses made between parent lines and the number of genotyped and phenotyped individuals within each cross are presented in the Supplementary data.

The comparison between MCQTL and MCQTL_LD was based on simulated data. Before the simulation, each design was analyzed by clusthaplo, and the local clustering obtained with a progression step of one cM was used both to simulate the QTL and for the MCQTL_LD analysis. A single biallelic QTL was simulated and a single chromosome was used for the comparative study. Given a QTL locus, we used the local clustering to assign to the parent lines belonging to the biggest class the mutated QTL allele; all other parent lines received the wild-type allele. In case of two or more biggest classes, we randomly chose one class. For each descendant family, we assigned to each descendant a QTL genotype given its parental alleles using a random draw that follows the probabilities of its QTL genotype given its markers. The phenotype of each descendant was simulated with a Gaussian law of mean 0 and variance 1 and an additive QTL effect linked to the mutated allele is added to the phenotype value. We simulated a single QTL at 100 loci on the densest chromosome of the descendant genetic map. We replicated each QTL locus 200 times. For each design, two values for the QTL mutated allele were used (0.15, 0.25 for Syngenta and Euralis2007; 0.25, 0.35 for Euralis2005). In total we analyzed 120,000 data sets with the two methods.

The QTL detection was conducted with the iQTLm method (Charcosset et al. 2001) using an additive QTL model. The 10 % detection threshold of each method was obtained using 5000 permutations (Doerge and Churchill 1996). The window length around the scan position to avoid spurious close QTL was set to 10 cM.

We calculated the precision of each method as the proportion of replicates where a detected QTL was located around the simulated QTL; replicates with no detected QTL were not included. We used four values for this surrounding interval (1, 2, 5, and 10 cM) and computed the precision for 100 simulated positions from the beginning of the chromosome. The *p* value of a one-side paired t-test between the precisions of the methods for the simulated positions was used to conclude for the significant superiority of MCQTL_LD in mapping QTL. We investigated the correlation of the difference in precision between MCQTL and MCQTL_LD at each QTL position with the fact of being on a marker or not, the empirical power of each method, the variability of the locus information at the putative QTL and the number of ancestral alleles which is proportional to the decrease in model parameters.

## Results

### Clusthaplo

Results for clusthaplo were obtained with the Euralis2007 data set that represents an intermediate design between a few families of large size and many families of small size. We investigated the impact of clusthaplo parameters on the average number of ancestral alleles and the number of changes in haplotype clustering (Table 1).

**Table 1:** Influence of the clusthaplo parameters on the average number of ancestor alleles and the number of clustering changes, defined by the change of at least one haplotype in the clustering result from locus to locus (373 clustering points in total)

Parameters	Default												
*w*_1_	Exp.	Exp.	Exp.	Exp.	Exp.	Exp.	Exp.	Laplace	Gauss	Unif.	Exp.	Laplace	Gauss
*w*_2_	Unif.	Unif.	Unif.	Unif.	Unif.	Unif.	Unif.	Unif.	Unif.	Unif.	1	1	1
Window length	20	15	10	5	1	20	20	20	20	20	20	20	20
Na.replace	True^*a*^	True	True	True	True	Na^*b*^	False^*c*^	True	True	True	True	True	True
Li and Jiang’s score, threshold by equilibrium sampling													
Nb alleles	4.25	4.89	5.52	5.91	3.70	4.62	4.68	4.50	4.43	4.15	4.66	4.70	4.68
Nb changes	138	155	135	143	175	131	133	143	146	144	163	154	162
Extended Li and Jiang’s score, threshold by equilibrium sampling													
Nb alleles	4.14	4.52	4.22	4.55	4.17	4.50	4.51	4.40	4.33	4.04	4.61	4.64	4.64
Nb changes	138	168	177	220	198	127	130	148	143	140	159	152	154

^*a*^Comparison of alleles involving missing data gives always a true result

^*b*^Comparison of alleles involving missing data gives a non-assigned result

^*c*^Comparison of alleles involving missing data gives a false result

As expected, the window length had the strongest impact on the clustering result. Other parameters only showed small differences. It is difficult to highlight a clear impact of the window length. However, the smaller was the length, the less stable was the clustering along the genome. Concerning the average number of ancestral alleles, the behavior of the Li and Jiang’s similarity score is simpler to explain than the extended one. With medium to large window, this average number decreased with the window length for the former. And it tends to the average marker alleles when the window becomes tiny. On the opposite, with the extended Li and Jiang’s similarity score, the average number of ancestral alleles fluctuated with the window length. And it tends to a number of ancestral alleles that is linked to the average relatedness when the window becomes tiny. The policies to handle missing marker data did not exhibit differences between non-assigned and false options for the allele comparisons. This was due to the marker data set which did not contain long segment of missing data. Indeed, when missing data occur isolated, the non-assigned and false options give the same similarity score. When the choice for na.replace was true, the average number of ancestral alleles decreased while the number of clustering increased. Between the *w*_1_ weight functions that have an exponential decay, the Exponential, Gaussian and Laplace functions ranged from the stronger clustering to the weaker when the *w*_2_ weight function was uniform. However, when the *w*_2_ weight function was constantly equal to 1, the above *w*_1_ weight functions did not show differences for the average of ancestor alleles and only small differences for the number of clustering changes. This is expected since, a 1 *w*_2_ weight is high compared to the Exponential, Gaussian and Laplace *w*_1_ weight, so these combinations give much more importance to the longest common segment compared to the alike-in-state alleles.

The extended Li and Jiang’s similarity score led to a decrease in average number of ancestral alleles compared to the non-extended score. The reason of this decrease is due to the Euralis2007 data set that is composed of highly related parent lines. The alike-in-state kinship on sparse windows tends to increase the similarity score for highly related haplotypes. On the contrary, it is an identity kinship matrix that models the independence for the computation of the threshold by the equilibrium sampling. This identity kinship matrix had an opposite effect on the similarity score compared to the Euralis2007 kinship matrix. Both effects were added to produce stronger clustering.

**Fig. 2:**
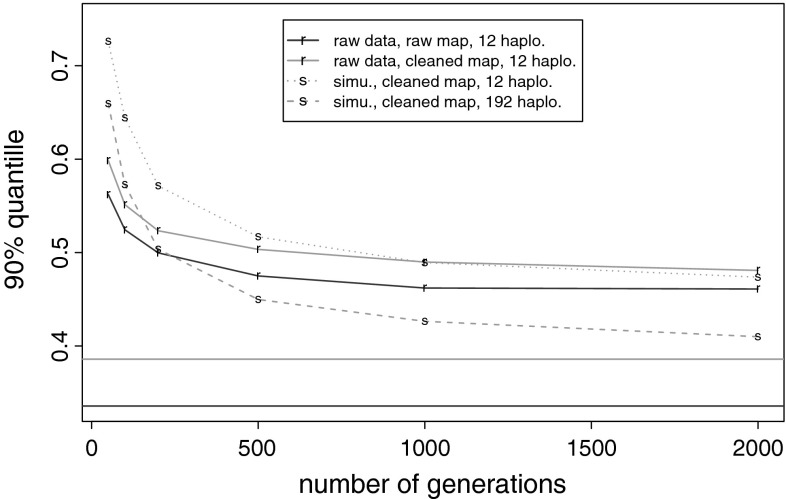
Comparison of the thresholds obtained by the mosaic and the equilibrium sampling. 90 % quantile obtained with the mosaic sampling (*labeled curves*) and the equilibrium sampling (*horizontal lines*). Samplings on the Euralis2007 raw data and raw map (*red full line*), samplings on the Euralis2007 raw data using a map cleaned of markers located at the same positions and monomorphic markers (*green full line*), samplings on simulated population of 12 related haplotypes and the cleaned map (*green pointed line*), samplings on a simulated population of 192 related haplotypes and the cleaned map (*green dashed line*) (color figure online)

We then analyzed the difference between the threshold computation methods. It is clear that the equilibrium sampling gives the smallest threshold and thus increases the clustering, since the equilibrium between markers and the equal allele frequencies are assumptions that produce the most entropic situation. The mosaic sampling is more adapted to the haplotype data set. Its behavior is illustrated by Fig. 2. We showed that the mosaic threshold converges to a limit when the number of generations of the mosaic sampling increased. This limit was attained with about 500 generations in the Euralis2007 design. We also showed that the equilibrium and the mosaic thresholds became close together with increasing generations, when the size of the initial haplotype population is large enough and the map is cleaned of non polymorphic markers and markers located at the same position. Indeed, these above markers could not be mixed up by the mosaic sampling, so they induced differences between the equilibrium and the mosaic thresholds. This is highlighted in Fig. 2, by the increasing of the initial haplotype population size (from 12 to 192). We simulated a F5 population which is a population of strong relatedness and we sampled 6 or 96 diploid individuals to create the initial haplotype population. With 12 haplotypes, the mosaic threshold did not converge to the equilibrium threshold whereas with 192 haplotypes the equilibrium and the mosaic thresholds became close together with increasing generations in the mosaic sampling.

**Fig. 3:**
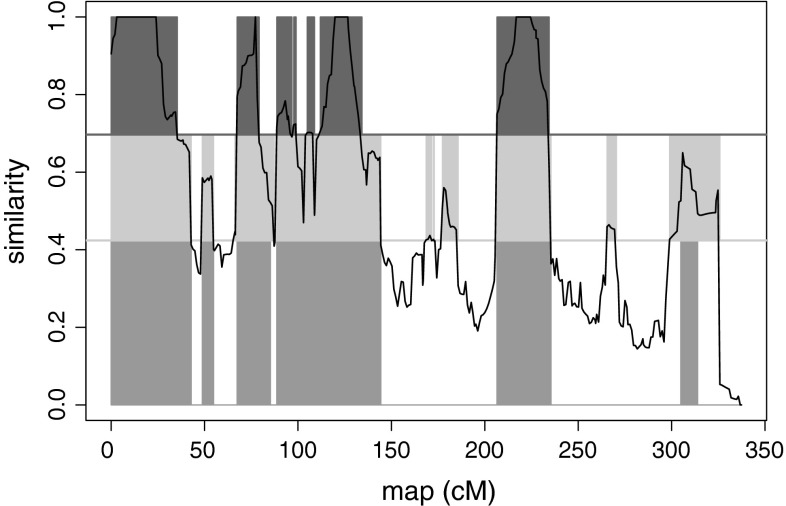
Comparison of the clustering of haplotypes obtained by the HMM and the mosaic and equilibrium thresholds. Clusterings of a haplotype pair of the Euralis2007 design, by the HMM (in *red*), a 95 % quantile by the equilibrium sampling (in *green*) and a 95 % quantile by the mosaic sampling of 200 generations (in *blue*). The chromosome blocs are colored where the haplotypes are clustered, the *black curve* is the similarity signal, the *horizontal green line* is the threshold estimated by an equilibrium sampling and the *blue* one was obtained by a mosaic sampling (color figure online)

Figure 3 illustrates the difference between the three clustering methods on an haplotype pair. Although we observed an overall consistency, it is clear that HMM method tends to make longer shared segments than equilibrium and mosaic methods. This was confirmed by 91.2 clustering changes in average over the parameter cases studied in Table 1 (limited to a window length of 20 cM and for the extended score) compared to 143.4 for the equilibrium clustering method. Adding that the HMM method is much faster to compute, it should be the chosen clustering method for its stability. However, we do not chose it as the default method to limit the dependency of our package to another R package since we used the RHmm package (http://www.r-project.org, http://r-forge.r-project.org/projects/rhmm/) to estimate the HMM models and to cluster the haplotypes. The second reason is that RHmm has huge difficulty to estimate the HMM models when the window length is small since the similarity score fluctuates too much and thus is far from a HMM signal in that cases.

### MCQTL_LD

The comparison between MCQTL and MCQTL_LD was based on 120,000 simulated data sets: 100 QTL loci, each with 200 replications, two values for the QTL mutated allele for each design (0.15, 0.25 for Syngenta and Euralis2007; 0.25, 0.35 for Euralis2005).

The parent clustering gave in average 3.39 ancestral alleles for four parent lines with the Syngenta data set which was coherent to the pedigree information we got. Indeed, only two of the four parent lines share a close ancestor. The Euralis designs which contained more related parent lines gave very different numbers: 5.83 (5.93) ancestral alleles for 12 (16) parent lines with the Euralis2007 (respectively, Euralis2005).

The thresholds of the 10 % type I error, obtained by 5,000 permutations, were equal to 2.47, 2.27, and 2.12, respectively, for Syngenta, Euralis2007, and Euralis2005 designs using MCQTL. They increased, respectively, to 2.54, 2.64, and 2.64 using MCQTL_LD. This is due to a test process that has a less autocorrelation function with MCQTL_LD compared to that of MCQTL. Indeed, the change in the number of parameters from locus to locus in MCQTL_LD has a tendency to lessen the correlation between consecutive tests. So, as the Bonferroni correction increases with the number of independent tests, the genome-wide threshold increases in MCQTL_LD.

Table 2 presents the precision averaged over the simulated positions and the *p* value of one-side paired t-test between the precisions of the two methods as well as the detection power. The results clearly showed that there was a gain both in precision and power as we analyzed the data with MCQTL_LD instead of MCQTL. This gain was limited while significant in a design with a small number of large families but was high in a design with a large number of small families. The highest gains were for surrounding intervals of 2 and 5 cM. The increase was around 8 % points for a surrounding interval of 2 cM in the Syngenta design and jumped to 25 % points for a surrounding interval of 5 cM in the Euralis2005 design.

**Table 2:** Average precision of MCQTL and MCQTL_LD as the average over 100 simulated positions of the proportion of replicates where a detected QTL was located around the simulated QTL given that at least one QTL was detected, for a surrounding interval of 1, 2, 5 and 10 cM

Design	Allele value	Method	1 cM	2 cM	5 cM	10 cM	Power^*a*^
Syngenta	0.15	MCQTL	11.14	22.89	46.24	62.79	41.23
		MCQTL_LD	15.08	28.81	51.89	66.36	43.50
		*P*val^*b*^	9 × 10^−5^	1 × 10^−8^	1 × 10^−7^	2 × 10^−5^	2 × 10^−5^
	0.25	MCQTL	20.61	41.53	72.50	88.09	88.13
		MCQTL_LD	26.84	49.44	78.09	89.57	89.30
		*P*val	2 × 10^−5^	3 × 10^−9^	3 × 10^−8^	2 × 10^−3^	1 × 10^−3^
Euralis2007	0.15	MCQTL	4.47	9.76	22.77	38.04	22.48
		MCQTL_LD	8.14	16.98	36.49	50.39	26.17
		*P*val	5 × 10^−4^	4 × 10^−7^	7 × 10^−18^	2 × 10^−17^	5 × 10^−12^
	0.25	MCQTL	8.26	20.57	45.12	67.71	59.14
		MCQTL_LD	17.62	33.61	62.84	78.37	68.08
		*P*val	2 × 10^−7^	5 × 10^−10^	9 × 10^−21^	2 × 10^−18^	9 × 10^−24^
Euralis2005	0.25	MCQTL	4.96	11.10	26.00	44.81	43.51
		MCQTL_LD	13.87	26.87	48.21	64.29	57.43
		*P*val	4 × 10^−8^	1× 10^−12^	3 × 10^−25^	1× 10^−29^	1× 10^−33^
	0.35	MCQTL	7.03	16.77	38.85	62.87	75.35
		MCQTL_LD	21.48	39.12	64.31	80.04	85.66
		*P*val	2 × 10^−10^	4 × 10^−15^	2 × 10^−26^	6 × 10^−27^	8 × 10^−26^

^*a*^Average of the detection power of each method over 100 simulated positions

^*b*^ *p* value of a one-side paired *t* test for the significant superiority in precision and power of MCQTL_LD

**Fig. 4:**
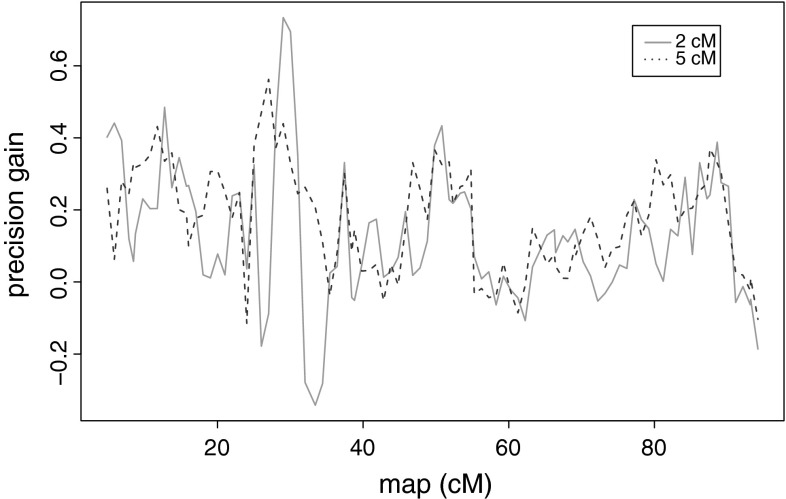
Variability along the chromosome of the precision gain of MCQTL_LD over MCQTL. Precision gain of MCQTL_LD over MCQTL for the Euralis2007 design and a value of the mutated allele of 0.25, for a surrounding interval of 5 cM (*red dashed line*), for a surrounding interval of 2 cM (*green full line*) (color figure online)

Table 2 presents average results that hide a high variation from position to position. This fact is illustrated by Fig. 4 where the precision gain of MCQTL_LD over MCQTL is plotted for the Euralis2007 design and a value of the mutated allele of 0.25 (similar behaviors were obtained for the other designs and other mutated allele values). It was then of interest to find the main features that could explain this high variability over positions of the precision gain. We investigated the correlation of the precision gain at each position with the fact of being on a marker or not, the empirical power, the variability of the locus information at the putative QTL and the number of ancestral alleles which is proportional to the decrease in model parameters. The number of ancestral alleles was a significant explanation for both the gain in power and the gain in precision (see Table 3). However, none of the other features were correlated to the above gains for small surrounding intervals (1 and 2 cM) and there was no clear picture for the other surrounding intervals and the gain in power (see Supplementary data).

**Table 3:** Pearson correlation (and its *p* value) of the number of ancestral alleles and the gain of MCQTL_LD over MCQTL, for the precision within a surrounding interval of 1, 2, 5, and 10 cM and the power

Design	Value of the mutated allele	1 cM	2 cM	5 cM	10 cM	Power
Syngenta	0.15	0.49 (1.9 × 10^−7^)	0.58 (3.2 × 10^−10^)	0.68 (4.4 × 10^−15^)	0.65 (2.9 × 10^−13^)	0.83 (5.1× 10^−27^)
	0.25	0.39 (4.4 × 10^−5^)	0.40 (3.7 × 10^−5^)	0.54 (5.8 × 10^−9^)	0.48 (4.1 × 10^−7^	0.78 (2.4 × 10^−21^)
Euralis2007	0.15	0.43 (6.8 × 10^−6^)	0.37 (2.2 × 10^−4^)	0.29 (2.8 × 10^−3^)	0.46 (1.9 × 10^−6^)	0.39 (5.7 × 10^−5^)
	0.25	0.37 (1.7 × 10^−4^)	0.29 (3.9 × 10^−3^)	0.21 (3.6 × 10^−2^)	0.50 (1.7 × 10^−7^)	0.57 (1.1 × 10^−9^)
Euralis2005	0.25	0.26 (1.1 × 10^−2^)	0.34 (9.8 × 10^−4^)	0.22 (3.7 × 10^−2^)	0.36 (3.9 × 10^−4^)	0.63 (1.2 × 10^−11^)
	0.35	0.21 (4.3 × 10^−2^)	0.28 (6.8 × 10^−3^)	0.17 (1.0 × 10^−1^)	0.30 (4.1 × 10^−3^)	0.37 (3.4 × 10^−4^)

## Discussion

We have presented a method and an R package, named clusthaplo. Its aim is to use a pairwise similarity measure locally along the genome. This measure reflects the alike-in-state status of alleles and the length of the longest common segment to cluster the haplotypes and to assign to each haplotype a probable ancestral allele corresponding to its class in the clustering. Clusthaplo outputs are readable by MCQTL and the ancestral alleles are plugged in the model replacing the parental alleles. Hence, the number of model parameters is lessened and a significant boost in the detection accuracy is achieved.

The pairwise similarity measure is based on the one proposed by Li and Jiang (2005). Its flexibility is allowed by the use of two weight functions, one for the alike-in-state allele comparison and one for the longest common segment. As the genetic population theory predicts an exponential decay of the linkage disequilibrium per generation, we have implemented in clusthaplo three different functions with exponential decay, an Exponential, Gaussian and Laplace function. These functions should be used in the computation of the alike-in-state part of the similarity score since they are adapted to the decay of linkage disequilibrium and so could not put strong weight on a allele that appears to be identical in two haplotypes by chance or by genotyping errors. The two other weight functions give the same weight (1 or a constant different to 1 for the Uniform function) to all the marker loci. They should be used to the longest common segment part of the similarity score to reflect the length of this shared segment. We showed that the choice of the weight functions led to few differences in clustering. We implemented by default Exponential and Uniform functions as they led to one of the strongest and more stable clustering of the haplotypes.

We extended the similarity measure of Li and Jiang (2005) to increase its reliability when computed with few markers by making use of known information on the haplotype relatedness as the kinship coefficients. By default, the kinship coefficients are computed by the average of the alike-in-state alleles with all the marker information. We showed with a highly related population that this extended similarity measure led to a stronger clustering compared to the Li and Jiang’s one.

The pairwise similarity measure is computed by sliding a window along the genome. We showed that the length of the window has a strong impact on the clustering. This length should be chosen regarding the relatedness of the haplotypes. A highly related population is known to share long haplotype blocks, so the window should span large segments to exhibit local differences in the genome. On the opposite, independent haplotypes should be studied with smaller window length.

At each scan locus, haplotypes are assigned a class by the transitive closure of a weighted filtered graph whose nodes are the haplotypes and weights, the pairwise similarities. Two methods are proposed to filter the graph; a threshold computed by simulations of nearly independent haplotypes to control the risk of false link between haplotypes or a fitted HMM. We showed that the HMM method is more stable in the clustering leading to longer common blocks than the threshold method. Two sampling strategies are implemented to compute a threshold, the equilibrium and the mosaic sampling. The mosaic sampling depends on a parameter that should be chosen around 500 to achieve convergence. We showed that the equilibrium sampling gives always a smaller threshold value than the mosaic sampling as the equilibrium sampling mimics the most entropic situation with independent and equifrequent bi-allelic markers. This threshold value should be preferred if the goal is to cluster the haplotypes as much as possible.

The current implementation of MCQTL_LD was developed to analyze usual type of populations derived from two inbred lines such as BC, HD, F2 to F7 families or RIL populations. For each parent lines of the multi-cross design, clusthaplo outputs an assignation to a class or what we call an ancestral allele per scan locus. This 0-1 assignation is not necessary for the marker regression model and its plug-in function. It is possible to feed MCQTL_LD with a smoother function, like for instance the local IBD probabilities of having received each ancestral alleles.

When using clusthaplo with *w*_1_ = 1, *w*_2_ = 0 and a threshold close to 1, the clustering finds the different haplotypes present within the sliding window. So with this rough choice, MCQTL_LD model is a multi-allelic QTL with one parameter per local haplotypes as was proposed by Jansen et al. (2003). Other choices of the weight functions and clustering methods lead to cluster the local haplotypes regarding their pairwise similarities and lead to the ancestral allele model. We can also imagine reducing the window length to get only one locus in it. With this type of tiny window and only SNP markers, bi-allelic QTLs are modeled by MCQTL_LD leading to a joint linkage association model with by-family means to model the population structure and linkage marker regression coefficients at the SNP loci. Moreover, the so-called disconnected model in MCQTL (Jourjon et al. 2005) is the homoscedastic linear approximation of the joint linkage model used to analyze the NAM population (Yu et al. 2008; Li et al. 2011). It is the least parsimonious with intra-family QTL parameters. The clusthaplo clustering plug-in allows a wide type of joint linkage and association models. The association model can be as simple as the SNP association model or a little more complex when taking into account all the different local haplotypes or the local ancestral alleles. So MCQTL and MCQTL_LD, both together, can analyze for any multi-population design a large extent of joint linkage and association models. The importance of having different complementary models to analyze complex traits was clearly shown by Bardol et al. (2013).

MCQTL_LD offers a QTL model featuring a versatility that is not found in other application softwares. The marker regression model, implemented in MCQTL_LD, has the general properties of linear model, which include the robustness to non linear residuals and it was proved to be asymptotically equivalent to the QTL mixture model (Rebai et al. 1995). This model benefits of the robust method of permutation tests (Doerge and Churchill 1996). Finally, dominance parameters and epistasis QTLs can be included in the model. These interactions QTL effects are already implemented in MCQTL and can be analyzed more precisely in MCQTL_LD thanks to the clusthaplo plug-in.

## Conclusion

MCQTL_LD and clusthaplo are unique software tools that permits to analyze multiple related families to detect and localize QTL. They implement a QTL mapping method that makes use of both linkage and linkage disequilibrium. The linkage disequilibrium is taking into account by clusthaplo that clusters the family parents and assigns to each of them at each position along the genome a probable ancestral allele. MCQTL_LD uses these probable ancestral alleles to lessen the number of its model parameters.

Clusthaplo uses a pairwise similarity measure computed in a sliding window along the genome to cluster the parent haplotypes. This measure reflects the alike-in-state status of alleles and the length of the longest common segment. We have implemented different options to compute this similarity measure. We showed that the window length has a strong impact on the clustering and gave insights to chose this length.

We carried out intensive simulations on three real genetic data sets, that represent three contrasted designs from a small number of large-size families to a huge number of medium-/small-size families. We showed that MCQTL_LD outperforms the classical linkage mapping analysis of MCQTL. The maximum gain in power and in accuracy was obtained for the design with a huge number of medium-/small-size families. In that design, the number of detected QTL in a narrow interval of 2 cM around the simulated QTL was more than doubled.

## Electronic supplementary material

Below is the link to the electronic supplementary material.

PDF (347 KB)

## References

[CR1] Bardol N, Ventelon M, Mangin B, Jasson S, Loywick V, Couton F, Derue C, Blanchard P, Charcosset A, Moreau L (2013) Combined linkage and linkage disequilibrium QTL mapping in multiple families of maize (*Zea mays* L.) line crosses highlights complementarities between models based on parental haplotype and single locus polymorphism. Theor Appl Genet 126(11):2717–2736. doi:10.1007/s00122-013-2167-9 10.1007/s00122-013-2167-9 23975245

[CR2] Bink MCAM, Uimari P, Sillanpaa MJ, Janss LLG, Jansen RC (2002). Multiple QTL mapping in related plant populations via a pedigree-analysis approach. Theor Appl Genet.

[CR3] Bink MCAM, Totir LR, ter Braak CJF, Winkler CR, Boer MP, Smith OS (2012). QTL linkage analysis of connected populations using ancestral marker and pedigree information. Theor Appl Genet.

[CR4] Blanc G, Charcosset A, Mangin B, Gallais A, Moreau L (2006). Connected populations for detecting quantitative trait loci and testing for epistasis: an application in maize. Theor Appl Genet.

[CR5] Cadic E, Coque M, Vear F, Grezes-Besset B, Pauquet J, Piquemal J, Lippi Y, Blanchard P, Romestant M, Pouilly N, Rengel D, Gouzy J, Langlade N, Mangin B, Vincourt P (2013). Combined linkage and association mapping of flowering time in sunflower (*Helianthus annuus* L.). Theor Appl Genet.

[CR6] Charcosset A, Mangin B, Moreau L, Combes L, Jourjon MF, Gallais A (2001) Heterosis in maize investigated using connected RIL populations. In: Gallais A, Dillmann C, Goldringer I (eds) Quantitative genetics and breeding methods: the way ahead, Colloques de l INRA, vol 96, pp 89–98

[CR7] Crepieux S, Lebreton C, Flament P, Charmet G (2005). Application of a new IBD-based QTL mapping method to common wheat breeding population: analysis of kernel hardness and dough strength. Theor Appl Genet.

[CR8] Doerge RW, Churchill GA (1996). Permutation tests for multiple loci affecting a quantitative character. Genetics.

[CR9] Farnir F, Grisart B, Coppieters W, Riquet J, Berzi P, Cambisano N, Karim L, Mni M, Moisio S, Simon P, Wagenaar D, Vilkki J, Georges M (2002). Simultaneous mining of linkage and linkage disequilibrium to fine map quantitative trait loci in outbred half-sib pedigrees: revisiting the location of a quantitative trait locus with major effect on milk production on bovine chromosome 14. Genetics.

[CR10] Fournier-Level A, Wilczek AM, Cooper MD, Roe JL, Anderson J, Eaton D, Moyers BT, Petipas RH, Schaeffer RN, Pieper B, Reymond M, Koornneef M, Welch SM, Remington DL, Schmitt J (2013). Paths to selection on life history loci in different natural environments across the native range of arabidopsis thaliana. Mol Ecol.

[CR11] Gilmour AR, Thompson R, Cullis BR (1995). Average information REML: an efficient algorithm for variance parameter estimation in linear mixed models. Biometrics.

[CR12] Haley CS, Knott SA (1992). A simple regression method for mapping quantitative trait loci in line crosses using flanking markers. Heredity.

[CR13] Jansen RC (1994). Controlling the type i and type ii errors in mapping quantitative trait loci. Genetics.

[CR14] Jansen RC, Jannink JL, Beavis WD (2003). Mapping quantitative trait loci in plant breeding populations: use of parental haplotype sharing. Crop Sci.

[CR15] Jourjon MF, Jasson S, Marcel J, Ngom B, Mangin B (2005). MCQTL: multi-allelic QTL mapping in multi-cross design. Bioinformatics.

[CR16] Kao CH, Zeng ZB, Teasdale RD (1999) Multiple interval mapping for quantitative trait loci. Genetics 152(3):1203–1216. http://www.genetics.org/content/152/3/1203.abstract, http://www.genetics.org/content/152/3/1203.full.pdf+html 10.1093/genetics/152.3.1203 PMC1460657 10388834

[CR17] Kover PX, Valdar W, Trakalo J, Scarcelli N, Ehrenreich IM, Purugganan MD, Durrant C, Mott R (2009). A multiparent advanced generation inter-cross to fine-map quantitative traits in *Arabidopsis thaliana*. PLoS Genet.

[CR18] Lagunes Espinoza LD, Julier B (2013). Qtl detection for forage quality and stem histology in four connected mapping populations of the model legume Medicago truncatula. Theor Appl Genet.

[CR19] Lander ES, Botstein D (1989). Mapping mendelian factors underlying quantitative traits using rflp linkage maps. Genetics.

[CR20] Lariepe A, Mangin B, Jasson S, Combes V, Dumas F, Jamin P, Lariagon C, Jolivot D, Madur D, Fievet J, Gallais A, Dubreuil P, Charcosset A, Moreau L (2012). The genetic basis of heterosis: multiparental quantitative trait loci mapping reveals contrasted levels of apparent overdominance among traits of agronomical interest in maize (*Zea mays* L.). Genetics.

[CR21] Li H, Bradbury P, Ersoz E, Buckler ES, Wang J (2011) Joint QTL Linkage Mapping for Multiple-Cross Mating Design Sharing One Common Parent. PLOS ONE 6(3). doi:10.1371/journal.pone.0017573 10.1371/journal.pone.0017573 PMC3057965 21423655

[CR22] Li J, Jiang T (2005). Haplotype-based linkage disequilibrium mapping via direct data mining. Bioinformatics.

[CR23] Meuwissen THE, Karlsen A, Lien S, Olsaker I, Goddard ME (2002). Fine mapping of a quantitative trait locus for twinning rate using combined linkage and linkage disequilibrium mapping. Genetics.

[CR24] Moreau D, Burstin J, Aubert G, Huguet T, Ben C, Prosperi JM, Salon C, Munier-Jolain N (2012). Using a physiological framework for improving the detection of quantitative trait loci related to nitrogen nutrition in Medicago truncatula. Theor Appl Genet.

[CR25] Pauly L, Flajoulot S, Garon J, Julier B, Beguier V, Barre P (2012). Detection of favorable alleles for plant height and crown rust tolerance in three connected populations of perennial ryegrass (*Lolium perenne* L.). Theor Appl Genet.

[CR26] Perez-Enciso M (2003). Fine mapping of complex trait genes combining pedigree and linkage disequilibrium information: a Bayesian unified framework. Genetics.

[CR27] R Development Core Team (2008) R: A Language and Environment for Statistical Computing. R Foundation for Statistical Computing, Vienna, Austria, http://www.R-project.org (ISBN 3-900051-07-0)

[CR28] Rebai A, Goffinet B (1993). Power of tests for QTL detection using replicated progenies derived from a diallel cross. Theor Appl Genet.

[CR29] Rebai A, Goffinet B, Mangin B (1995). Comparing power of different methods for QTL detection. Biometrics.

[CR30] Xie CQ, Gessler DDG, Xu SZ (1998). Combining different line crosses for mapping quantitative trait loci using the identical by descent-based variance component method. Genetics.

[CR31] Yi NJ, Xu SZ (2001). Bayesian mapping of quantitative trait loci under complicated mating designs. Genetics.

[CR32] Yu J, Pressoir G, Briggs WH, Bi IV, Yamasaki M, Doebley JF, McMullen MD, Gaut BS, Nielsen DM, Holland JB (2005). A unified mixed-model method for association mapping that accounts for multiple levels of relatedness. Nat Genet.

[CR33] Yu J, Holland JB, McMullen MD, Buckler ES (2008). Genetic design and statistical power of nested association mapping in maize. Genetics.

