# Supplementary material for: Clusthaplo: a plug-in for MCQTL to enhance QTL detection using ancestral alleles in multi-cross design
Source: Theor Appl Genet. 2014 Jan 31;127(4):921–33. doi: 10.1007/s00122-014-2267-1 (PMC3964294; doi:10.1007/s00122-014-2267-1)
Supplement: Supplementary file 1 — PDF (347 KB) [file 122_2014_2267_MOESM1_ESM.pdf]

TAG manuscript No.
(will be inserted by the editor)

Clusthaplo: a plug-in for MCQTL to enhance QTL detection using ancestral alleles in multi-cross design - Supplementary tables and figure

Damien Leroux · Abdelaziz Rahmani · Sylvain Jasson · Marjolaine Ventelon · Florence Louis · Laurence Moreau · Brigitte Mangin

Received: date / Accepted: date

Damien Leroux
INRA, UR875, Unité de Mathématique et Informatique Appliquées de Toulouse, Chemin de Borde Rouge, F-31326 Castanet-Tolosan, France
Tel.: +33561285493
Fax: +33561285335
E-mail: Damien Leroux - Damien.Leroux@toulouse.inra.fr

Abdelaziz Rahmani
INRA, UR875, Unité de Mathématique et Informatique Appliquées de Toulouse, Chemin de Borde Rouge, F-31326 Castanet-Tolosan, France
E-mail: Abdelaziz Rahmani - rahmani.abdelaziz@gmail.com

Sylvain Jasson
INRA, UR875, Unité de Mathématique et Informatique Appliquées de Toulouse, Chemin de Borde Rouge, F-31326 Castanet-Tolosan, France
E-mail: Sylvain Jasson - sylvain.jasson@toulouse.inra.fr

Marjolaine Ventelon
EURALIS SEMENCES, Service Biométrie, Domaine de Sandreau, 31700 MONDONVILLE, France
E-mail: Marjolaine Ventelon - marjolaine.ventelon@euralis.fr

Florence Louis
Syngenta Seeds, 12 chemin de l'Hobit, 31790 Saint-Sauveur, France
E-mail: Florence Louis - florence.louis@syngenta.com

Laurence Moreau
INRA, UMR 0320 / UMR 8120 Genet Vegetale, Ferme du Moulon F-91190 Gif Sur Yvette, France
E-mail: Laurence Moreau - moreau@moulon.inra.fr

Brigitte Mangin
INRA, UR875, Unité de Mathématique et Informatique Appliquées de Toulouse, Chemin de Borde Rouge, F-31326 Castanet-Tolosan, France
E-mail: Brigitte Mangin - Brigitte.Mangin@toulouse.inra.fr

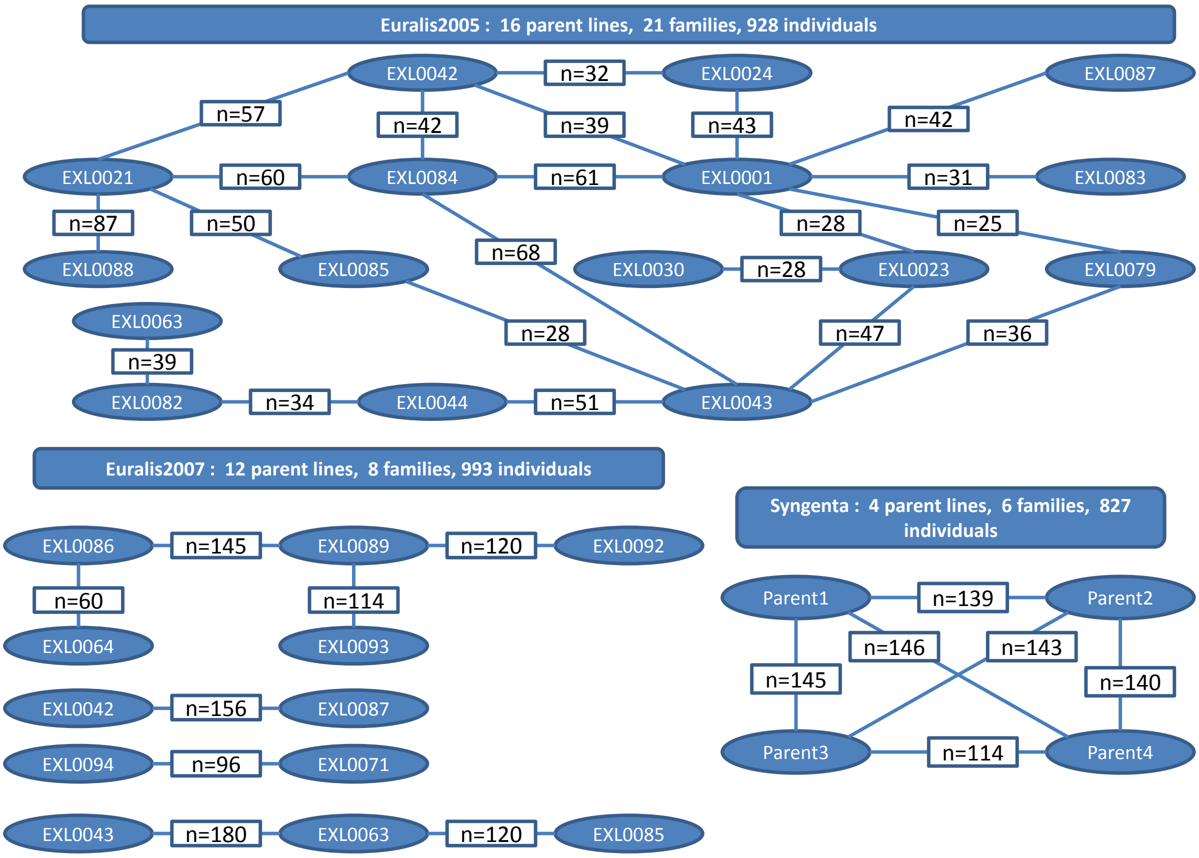

Fig. S1 Maize designs used by the simulations. The parent lines are represented by blue ovals. Those that were crossed are linked by a blue line, on which the white box gives the number of genotyped and phenotyped descendants.

Table S1: Pearson correlation of the power and the gain of MCQTL_LD over MCQTL, for the precision within a surrounding interval of 1 cM, 2 cM, 5 cM and 10 cM.

Design	value of the mutated allele	Method	average power	1 cM	2cM	5cM	10 cM
Syngenta							
	0.15	MCQTL	41.23	-0.05	-0.13	-0.02	-0.03
		MCQTL_LD	43.50	0.14	0.11	0.26**	0.33***
	0.25	MCQTL	88.13	-0.02	-0.21	-0.06	0.11
		MCQTL_LD	89.30	0.11	-0.02	0.16	0.30 **
Euralis2007							
	0.15	MCQTL	22.48	-0.10	-0.09	-0.08	-0.06
		MCQTL_LD	26.17	-0.06	0.001	0.25 *	0.25 *
	0.25	MCQTL	59.14	-0.21	-0.18	-0.09	-0.32 **
		MCQTL_LD	68.08	-0.13	-0.13	0.01	-0.13
Euralis2005							
	0.25	MCQTL	43.51	0.01	0.08	0.37***	0.53 ***
		MCQTL_LD	57.42	0.03	0.15	0.38 ***	0.59 ***
	0.35	MCQTL	75.35	-0.06	-0.04	0.10	0.18
		MCQTL_LD	85.66	-0.04	0.02	0.09	0.17

* for $0.05 <$ p-value ≤ 0.01, ** for $0.01 <$ p-value ≤ 0.001 and *** for $0.001 <$ p-value.

Table S2: Pearson correlation of "being on a marker" and the gain of MCQTL_LD over MCQTL, for the precision within a surrounding interval of 1 cM, 2 cM, 5 cM, 10 cM and the power.

Design	value of the mutated allele	1 cM	2cM	5cM	10 cM	power
Syngenta						
	0.15	-0.10	-0.19	-0.24 *	-0.06	0.08
	0.25	-0.12	-0.12	-0.30 **	-0.06	-0.13
Euralis2007						
	0.15	-0.03	0.007	0.04	0.04	0.08
	0.25	-0.13	-0.05	-0.006	0.14	0.20
Euralis2005						
	0.25	-0.03	0.09	0.08	0.14	0.08
	0.35	-0.06	0.07	-0.007	0.11	-0.02

* for $0.05 <$ p-value ≤ 0.01, ** for $0.01 <$ p-value ≤ 0.001 and *** for $0.001 <$ p-value.

Table S3: Pearson correlation of the variability of locus information and the gain of MCQTL_LD over MCQTL, for the precision within a surrounding interval of 1 cM, 2 cM, 5 cM, 10 cM and the power.

Design	value of the mutated allele	1 cM	2cM	5cM	10 cM	power
Syngenta						
	0.15	0.02	-0.04	0.02	0.10	0.21*
	0.25	-0.02	-0.14	-0.01	0.13	-0.06
Euralis2007						
	0.15	-0.14	-0.10	0.006	-0.12	0.16
	0.25	-0.12	-0.08	-0.03	-0.34***	-0.09
Euralis2005						
	0.25	-0.012	0.06	0.38***	0.46***	0.10
	0.35	-0.09	-0.10	0.06	0.16	-0.80***

* for $0.05 <$ p-value ≤ 0.01, ** for $0.01 <$ p-value ≤ 0.001 and *** for $0.001 <$ p-value.

The variability of locus information was calculated following

$$\sum_d 1_{\{QTL^l(P1) \neq QTL^l(P2); d=P1P2\}} \sum_n (X^l_{dn} - X^l_{d.})^2$$

where l denotes a locus, d denotes a descendant family, P_1 P_2 denote the two parent lines of d, n denotes the individuals, $X^l_{dn} = 2 * Z^l_{dnP1} - 1$ with Z^l_{dnP1} is the probability of the nth individual of descendant family d having the homozygous genotype $P1P1$ at locus l given its marker information and $X^l_{d.}$ is the X^l_{dn} mean of d, 1_x equals 1 if x is true and 0 otherwise and $QTL^l(P1)$ (resp.$QTL^l(P2)$) denotes the QTL allele of parent line $P1$ (resp. $P2$) at locus l.
